# Clinical effect and follow-up of laparoscopic radical proximal gastrectomy for upper gastric carcinoma

**DOI:** 10.3389/fonc.2023.1167177

**Published:** 2023-03-29

**Authors:** Wei Meng, Huang Ya-di, Cao Wei-bo, Zhao Ru-dong, Cheng Ze-wei, Jun Ou Yang, Yan Ze-peng, Chen Chuan-qi, Liang Yi-ze, Sun Dan-ping, Yu Wen-bin

**Affiliations:** ^1^ Department of Gastrointestinal Surgery Qilu Hospital of Shandong University, Jinan, China; ^2^ Department of General Surgery, Weihai Second Hospital, Weihai, China; ^3^ Department of General Surgery, Yangxin Hospital of Traditional Chinese Medicine, Yangxin, China

**Keywords:** upper gastric carcinoma, laparoscopic radical proximal gastrectomy, tubular esophagogastric anastomosis, traditional esophagogastrostomy, indocyanine green tracer technique

## Abstract

**Objective:**

To evaluate the safety and clinical effect of tubular esophagogastric anastomosis in laparoscopic radical proximal gastrectomy.

**Methods:**

A retrospective analysis was conducted involving 191 patients who underwent laparoscopic radical proximal gastrectomy in the Department of Gastrointestinal Surgery, Qilu Hospital of Shandong University from January 2017 to October 2020. Patients were divided into tubular esophagogastric anastomosis group (TG group) and traditional esophagogastric anastomosis group (EG group) according to the digestive tract reconstruction. Their intraoperative conditions, perioperative recovery and postoperative follow-up were compared. Patients were also divided into indocyanine green group and non-indocyanine green group according to whether or not indocyanine green tracer technology was used during the operation. Their intraoperative condition and perioperative recovery were compared and analyzed after propensity score matching.

**Results:**

The operation was successfully completed in all patients. Compared with the EG group, the TG group had less volume of gastric tube drainage, shorter gastric tube drainage time and proton pump inhibitors application time, and lower reuse rate of proton pump inhibitors. However, the TG group had a higher anastomotic stenosis at three months after surgery, as measured using anastomotic width and dysphagia score. Nevertheless, the incidence of reflux esophagitis and postoperative quality of life score in the TG group were lower compared with the EG group at 1st and 2nd year after surgery. In the indocyanine green analysis, the indocyanine green group had significantly shorter total operation time and lymph node dissection time and less intraoperative blood loss compared with the non-indocyanine green group. However, compared with the non-indocyanine green group, more postoperative lymph nodes were obtained in the indocyanine green group.

**Conclusion:**

Laparoscopic radical proximal gastrectomy is safe and effective treatment option for upper gastric cancer. Tubular esophagogastric anastomosis has more advantages in restoring postoperative gastrointestinal function and reducing reflux, but it has a higher incidence of postoperative anastomotic stenosis compared with traditional esophagogastrostomy. The application of indocyanine green tracer technique in laparoscopic radical proximal gastrectomy has positive significance.

## 1 Introduction

Gastric cancer is one of the most common malignant tumors of the digestive system, which ranks fifth in terms of incidence worldwide (1). Although the overall incidence of gastric cancer has shown a decreasing trend in recent years, the incidence of the upper gastric cancer is increasing, especially in East-Asian countries, such as China, South Korea, and Japan (2). At present, surgical resection remains the mainstream treatment for gastric cancer (3, 4). However, for the upper gastric cancer, especially adenocarcinoma at the esophagogastric junction, the optimal surgical method is still controversial (5). Japanese gastric cancer treatment guidelines (6) recommend radical proximal gastrectomy for early gastric cancer and radical total gastrectomy for advanced gastric cancer. Chinese gastric cancer treatment guidelines (7) recommend that the standard for proximal gastrectomy is ensuring adequate surgical margin and not limiting the stage of gastric cancer. Although total gastrectomy can reduce the risk of postoperative recurrence or metastasis can be reduced in patients, it may cause loss of gastric functions, such as digestion, absorption, and secretion, resulting in postoperative complications such as low hemoglobin, malnutrition, diarrhea and dumping syndrome (8). Compared with total gastrectomy, proximal gastrectomy is a simpler procedure that preserves partial gastric function, produces a treatment effect comparable with that of total gastrectomy, and can reduce the incidence of the aforementioned complications to a certain extent (9, 10). At present, proximal gastrectomy, especially traditional esophagogastrostomy, is widely accepted and used in clinical practice (11). However, its disadvantages such as acid reflux, heartburn, chest pain, anxiety and anorexia caused by long-term gastroesophageal reflux remain significant, as they negatively affect the quality of life of patients after surgery (12, 13). As a result, many scholars have explored and improved the digestive tract reconstruction methods after proximal gastrectomy to reduce the incidence of reflux esophagitis and improve the postoperative quality of life of patients (14). These methods include traditional esophagogastric anastomosis, tubular esophagogastric anastomosis, double muscle flap anastomosis, jejunal interposition anastomosis, and double-tract anastomosis. Among them, tubular esophagogastric anastomosis has demonstrated advantages such as simple preparation of tubular stomach and effective reduction of gastroesophageal reflux (15, 16). As such, it is widely accepted and applied in the clinic. However, its clinical efficacy has not been systematically evaluated. Therefore, we retrospectively analyzed and compared the clinical data of patients who received laparoscopic proximal gastrectomy + tubular esophagogastric anastomosis and traditional esophagogastrostomy from January 2017 to July 2020 at Qilu Hospital of Shandong University to evaluate the safety and clinical applicability of tubular esophagogastric anastomosis.

In addition, the emerging technology of indocyanine green (ICG) labeling and tracer technology has gained wide application in gastrointestinal surgery in combination with laparoscopic technology. Its can accurately identify tumors and improve the accuracy of lymph node dissection. Therefore, in this study, 191 patients were divided into ICG and non-ICG groups according to whether or not intraoperative ICG tracer technology was applied. Intraoperative condition, postoperative pathology and postoperative survival of patients in the two groups were compared and analyzed to explore the application value of ICG tracer technology in radical laparoscopic proximal gastrectomy.

## 2 Materials and methods

### 2.1 Patients (research object)

This study retrospectively collected the clinical data of 191 patients who underwent laparoscopic radical proximal gastrectomy in the department of gastrointestinal surgery, Qilu Hospital of Shandong University from January 2017 to October 2020. According to the type of digestive tract anastomosis, the patients were divided into tubular esophagogastric anastomosis group (TG group) and traditional esophagogastrostomy group (EG group). In addition, all patients were divided into ICG group and non-ICG group according to whether or not ICG tracer technique was used during the operation.

The inclusion criteria were as follows: (1) postoperative pathology confirmed gastric cancer and (2) laparoscopic radical proximal gastrectomy was performed.

The exclusion criteria were as follows: (1) patients who received antitumor therapy such as neoadjuvant chemotherapy, immunotherapy, or targeted therapy before surgery; (2) patients who underwent emergency surgery due to tumor perforation, bleeding, obstruction; (3) patients with with other primary malignant tumors; (4) patients with incomplete clinical or follow-up data.

This study complied with all ethical principles and was approved by the Ethics Committee of Qilu Hospital of Shandong University. All patients were informed of the risks associated with surgery in detail before surgery and signed informed consent.

### 2.2 Surgical procedure

All operations were performed by the gastrointestinal surgery team of Qilu hospital of Shandong University.

Lymph node dissection: All patients received D2 lymph node dissection. Briefly, the patient was placed in supine position and pneumoperitoneum was established using the 5-hole method. The tumor was then located by laparoscopy exploration of the abdominal cavity. Lymph nodes in groups 1, 2, 3, 4sa, 4sb, 7, 8, 9, 10 and 11d around the stomach were dissected, with those in groups 110 and 111 dissected using abdominal transhiatal approach, if necessary.

**Figure 1:**
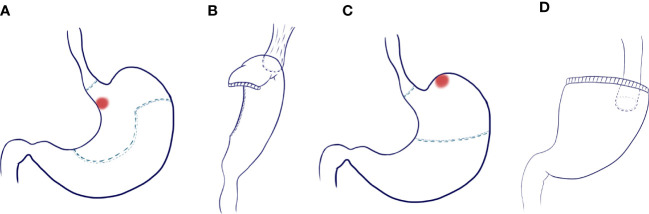
Scope of gastrectomy and reconstruction of digestive tract. **(A)** Excision range of tubular esophagogastric anastomosis and design of tubular stomach shape; **(B)** Esophago-tubular gastric anastomosis; **(C)** Scope of traditional esophagogastrostomy; **(D)** Traditional esophagogastrostomy.

Digestive tract reconstruction (**Figure 1**): After lymph node dissection under laparoscopy, an auxiliary incision about 5-7cm long was made in the median of the upper abdomen for all patients to complete digestive tract reconstruction under direct vision.

**Figure 2:**
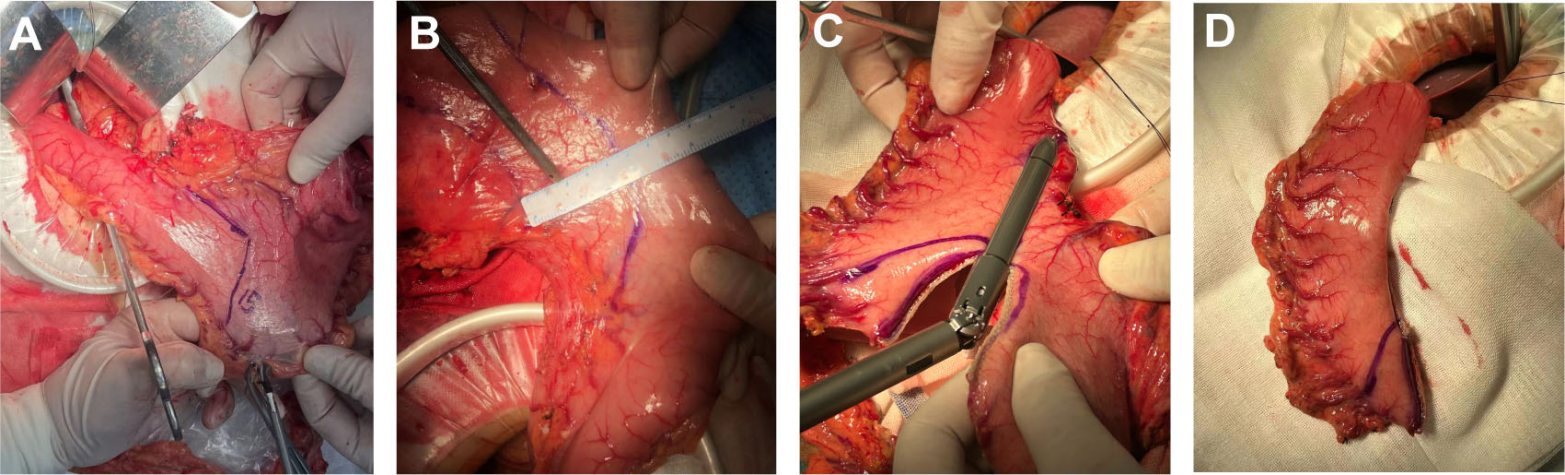
The procedural steps of tubular stomach operation. **(A)** Designing a tubular stomach shape. Lines were drawn from the pylorus 5cm above the pylorus along the minor curvature of the stomach to the side of the mouth, The residual stomach was designed to form a tubular structure about 15-20cm long and 4-5cm wide; **(B)** The tumor was located while ensuring that the tumor margin was greater than 5cm from the tubular gastric margin; **(C)** The tubular stomach was established according to the marks; **(D)** The shape of the tubular stomach.

Tubular esophagogastric anastomosis (**Figure 2**): The esophagus was severed 3 cm from the upper margin of the tumor, and the 25 mm round stapler base was placed. The shape of the residual stomach was designed according to the location and size of the tumor, such that the minimum distance between the proximal end of the residual stomach and the edge of the tumor was greater than 5 cm. About 5 cm above the pylorus, the lesser curvature of the stomach was excised according to the preset shape. The stomach was cut into a tubular shape with a width of about 4-5 cm and a length of 15-20 cm. The stomach was dissected 5 cm from the upper end of the gastric remnant, and the pylorus was mechanically expanded 3-4 times using oval forceps until relaxation. The stapler was placed from the incision, and the end-to-side anastomosis between the esophagus and large curved side of the tubular stomach was completed.

Traditional esophagogastrostomy: A similar method of stapler base placement was used as in tubular esophagogastric anastomosis. The stomach was transected by a straight line closure 5 cm from the lower margin of the tumor. The stump stomach at the upper end was opened, and the pylorus was mechanically expanded 3-4 times using oval forceps until relaxation. The stapler was placed from the incision, and the end-to-side anastomosis of esophagus and greater curvature of residual stomach was completed.

### 2.3 Postoperative recovery and follow-up

The removal time of gastric tube was based on the recovery of gastrointestinal function and the drainage volume of gastric tube. Typically, after the patient exhausts, the gastric tube can be removed when the drainage volume of the gastric tube is less than 100 ml in 24 hours and the color of the drainage fluid is normal. The time of the first drinking was determined by the patient’s gastric tube drainage and the general conditions of the patient during and after surgery. The pH of gastric fluid was measured at 8 a.m. every day before gastric tube removal. Mean value of the gastric fluid pH was recorded. Routine intravenous proton pump inhibitors (PPIs) was administered after surgery to all patients and discontinued after the first drinking. If patients reported acid reflux, heartburn or other symptoms during hospitalization, PPIs were reused. Before discharge, patients were smoothly exhaust, showed good tolerance to liquid diet and no significant abnormal fluctuation of body temperature, and reported no discomfort.

Patients were followed up for two years, including regular outpatient follow-up and telephone follow-up. Upper digestive tract angiography was performed three months after surgery to evaluate the anastomotic status and measure the anastomotic width. Abdominal CT examination and intensive scanning was performed every six months after surgery, whereas gastroscopy was reviewed every year after surgery. The frequency of follow-up was every three months after surgery, with tumor metastasis, recurrence or clinical death defined as the endpoint events. Tumor metastasis and recurrence were determined based on positive clinical symptoms, imaging examination or digestive endoscopy.

### 2.4 ICG injection and surgical procedures

Patients in the ICG group underwent gastroscope injection with ICG 16-24 hours before surgery. The ICG powder was dissolved and diluted to 0.625 mg/ml with sterile water. The tumor was injected in the proximal, distal, and bilateral position and labeled as follows: The injection needle was inserted into the submucosa of the stomach and marked in the order of 0.5 ml sterile water (lifting the mucosa) + 0.5 ml ICG solution + 0.5 ml sterile water (blocking to reduce ICG spilt). The near-infrared fluorescent imaging laparoscopy was used to evaluate ICG fluorescence. Since ICG can enter the lymphatic circulation and reach the lymph nodes around the stomach, the color of the lesion site and the lymph nodes around the stomach can be determined by switching the fluorescence mode during the operation (17). This technique was used to identify the lesion site and guide the dissection of lymph nodes.

### 2.5 Clinical data, postoperative recovery and follow-up

#### 2.5.1 Baseline characteristics of patients

Patient-level data were collected, including gender, age, body mass index (BMI), nutritional score (NRS), American Society of Anesthesiologists (ASA) physical status scores, chronic disease (such as hypertension, type 2 diabetes and coronary atherosclerotic heart disease), abdominal surgery history (such as cholecystectomy and appendectomy), the rate of ICG tracer technology usage, postoperative pathology, number of lymph nodes obtained and lymph node metastasis rate. The 7^th^ edition of the TNM classification by the Union for International Cancer Control/American Joint Committee on Cancer was used to evaluate the pathological data (18).

#### 2.5.2 Intraoperative indicators

Operation time, lymph node dissection time, digestive tract reconstruction time, blood loss were measured during the operation.

#### 2.5.3 Early postoperative recovery

Postoperative hospital stay, pain score (the first day after surgery), postoperative average daily drainage volume of the gastric tube and abdominal drainage tube, gastric drainage and abdominal drainage time, recovery time of intestinal function (first exhaust time and first drink time), average daily pH value of gastric fluid, application time and reuse rate of PPIs and early postoperative complications were measured. Early postoperative complications included pulmonary infection, pulmonary embolism, intraperitoneal bleeding, anastomosis-related complications (anastomotic fistula and anastomotic bleeding), cerebral infarction, atrial fibrillation and pyloric obstruction. Postoperative complications were graded using the Clavien-Dindo classification (19) (**Supplementary Table 1**).

#### 2.5.4 Long-term postoperative follow-up index

The rate of reflux esophagitis, quality of life, anastomotic width, dysphagia score, rate of anastomotic stenosis and living state were assessed. The quality of life of the patients was scored at 3 months and 1 year and 2 years after surgery, and the incidence of reflux esophagitis under gastroscopy was statistically analyzed 1 year and 2 years after surgery. The postoperative quality of life of the patients was scored using Gerd Q questionnaire (20, 21) (**Supplementary Table 2**). The questionnaire evaluated six dimensions of the quality of life of the patients: heartburn, acid reflux, nausea, epigastric pain, sleep status, and application of acid suppressors. Anastomotic width was measured using upper gastrointestinal angiography at 3 months, 1 year and 2 years after operation. Dysphagia score was also performed at 3 months, 1 year and 2 years after operation. Patients with anastomotic width less than 10 mm and dysphagia score more than 2 points were defined as having anastomotic stenosis and received endoscopic anastomotic balloon dilation. The dysphagia score (**Supplementary Table 3**) was applied to all patients based on the following five grades: 0: unrestricted for normal diet; 1: patients could eat solid food and felt mildly blocked when eating consciously; 2: patients could eat a semi-liquid diet; 3: patients could only eat a completely liquid diet; and 4: patients have difficulty with liquid diet.

### 2.6 Statistical analysis

SPSS (version 27.0) was used for statistical analysis. Continuous variable data were expressed as mean ± standard deviation (X ± s) and compared using independent sample t-test. Categorical variable data were compared using χ² test, Fisher test and the Mann–Whitney U test, with *p* < 0.05 considered statistically significant. Gender, age, BMI, NRS, ASA grade, chronic disease, abdominal surgery history and digestive tract reconstruction methods were used as matching variables in the ICG analysis. The 1:1 nearest neighbor matching method was used to match the ICG group and the non-ICG group, with the caliper value set to 0.02. P < 0.05 was considered statistically significant. Disease-free survival (DFS) and Overall survival (OS) were evaluated using the Kaplan-Meier method, and statistical differences between groups were evaluated using log-rank test.

## 3 Result

### 3.1 Baseline characteristics of patients

The baseline characteristics of patients in both groups are shown in **Table 1**. A total of 191 patients were included in this study, including 98 patients in the TG group and 93 patients in the EG group. There were no significant differences between the two groups in gender, age and other preoperative basic characteristics (*p* > 0.05). The ICG use rate in the TG group was higher (40% or 39/98) than that in the EG group (35% or 33/93), which was no significant difference between the two groups (*p* = 0.539). For postoperative pathology, 57 cases (58%) and 51 cases (55%) in the TG group and EG group were pathologically confirmed as early gastric cancer, respectively, with no significant difference in the depth of tumor invasion between the two groups (*p* > 0.05). The number of lymph nodes obtained after surgery and rate of lymph node metastasis were 32.59 ± 6.96 and 26% in the TG group and 31.59 ± 6.09 and 22% in EG group, respectively, with no significant statistical differences between the two groups (*p* > 0.05).

**Table 1:** Baseline patient’s characteristics.

	TG group	EG group	P value
	n = 98	n = 93	
Gender			0.165
male	78 (80%)	81 (87%)	
female	20 (20%)	12 (13%)	
Age (year)	63.66±7.39	64.29±7.31	0.556
BMI (kg/m²)	24.67±3.57	24.89±3.38	0.662
NRS			0.787
1	44 (45%)	43 (46%)	
2	30 (31%)	31 (33%)
> 2	24 (24%)	19 (20%)
ASA scores			0.112
I	2 (2%)	1 (1%)
II	87 (89%)	90 (97%)
III	9 (9%)	2 (2%)
chronic disease ^a^	49 (50%)	49 (53%)	0.71
abdominal surgery history ^b^	17 (17%)	11 (12%)	0.281
ICG rate	39 (40%)	33 (35%)	0.539
pT stage			0.643
1	57 (58%)	51 (55%)
2~3	41 (42%)	42 (45%)
pN stage			0.514
0	73 (74%)	73 (78%)
1~3	25 (26%)	20 (22%)
number of lymph nodes	32.59±6.96	31.59±6.09	0.293
number of positive lymph nodes	1.18±2.86	0.91±2.78	0.51

TG, tubular esophagogastric anastomosis; EG, traditional esophagogastric anastomosis.

BMI, body mass index; NRS, nutritional score; ASA, American Society of Anesthesiologists.

(a) Hypertension, type 2 diabetes, coronary atherosclerotic heart disease, etc.

(b) Cholecystectomy, appendectomy, etc.

pTNM staging was performed according to the AJCC 7^th^ edition.

### 3.2 Intraoperative indicators

The intraoperation indicators are shown in **Table 2**. The total operation time of the TG group and EG group was 239.73 ± 43.70 min and 233.55 ± 39.41 min, respectively. The digestive tract reconstruction time was 59.23 ± 7.37 min and 59.09 ± 7.77 min in the TG group and EG group, respectively. There were no significant differences in operation time, lymph node dissection time, digestive tract reconstruction time and intraoperative blood loss between the two groups (*p* > 0.05).

**Table 2:** Intraoperative indicators.

	TG group	EG group	P value
	n = 98	n = 93	
operation time (min)	239.73 ± 43.70	233.55 ± 39.41	0.306
lymph node dissection time (min)	115.77 ± 12.46	116.40 ± 13.64	0.738
digestive tract reconstruction time (min)	59.23 ± 7.37	59.09 ± 7.77	0.892
blood loss (ml)	44.08 ± 32.99	46.88 ± 31.21	0.548

### 3.3 Postoperative recovery

There were no significant difference between the two groups in the length of postoperative hospitalization (9.43 ± 2.72 vs. 10.19 ± 2.99, *p* = 0.066). Compared with the EG group, the TG group had lower mean postoperative gastric tube drainage volume (*p* < 0.001), shorter gastric drainage time (*p* < 0.001), and earlier first drinking time (*p* = 0.010). There were no significant differences between the two groups in postoperative pain score, first exhaust time, average drainage volume of abdominal drainage tube and abdominal drainage tube carrying time (*p* > 0.05). Compared with the EG group, the mean daily gastric fluid PH of the TG group was significantly higher (*p* = 0.022). The PPIs application time for the TG group was significantly lower than that for the EG group (5.46 ± 1.98 days vs. 6.64 ± 2.25 days, *p* = 0.002). Similarly, the PPIs reuse rate in the TG group was significantly lower than that in the EG group (6% vs. 23%) (*p* = 0.001). There was no significant difference in the occurrence of postoperative complications between the two groups. The specific postoperative recovery status is shown in **Table 3**.

**Table 3:** Postoperative recovery.

	TG group	EG group	P value
	n = 98	n = 93	
postoperative hospital stay (day)	9.43 ± 2.72	10.19 ± 2.99	0.066
pain score (first day)			0.787
2~3	31 (32%)	27 (29%)	
4~6	65 (66%)	65 (70%)	
7	2 (2%)	1 (1%)	
first exhaust time (hour)	76.45 ± 19.85	76.96 ± 19.60	0.859
first drinking time (hour)	121.03 ± 32.79	132.83 ± 29.24	0.010
average daily gastric drainage (ml)	52.41 ± 48.51	108.86 ± 91.67	<0.001
average daily abdominal drainage (ml)	96.70 ± 66.53	109.54 ± 91.41	0.267
gastric drainage time (hour)	102.11 ± 29.78	117.02 ± 28.26	<0.001
abdominal drainage time (day)	7.92 ± 1.89	7.91 ± 2.18	0.988
average daily PH value of gastric juice	6.31 ± 0.50	6.13 ± 0.59	0.022
PPIs application time (day)	5.46 ± 1.98	6.44 ± 2.25	0.002
reuse rate of PPIs	6 (6%)	21 (23%)	0.001
early complications	23 (23%)	24 (26%)	0.644
pulmonary infection	22 (22%)	21 (23%)	
bleeding	1 (1%)	2 (2%)	
anastomotic fistula	3 (3%)	2 (2%)	
cerebral infarction	1 (1%)	1 (1%)	
atrial fibrillation	0	1 (1%)	
pyloric obstruction	0	0	
Clavien-Dindo classification			0.967
I	3 (3%)	4 (4%)	
II	18 (18%)	18 (19%)	
III-IV	2 (2%)	2 (2%)	

PPIs, proton pump inhibitors.

A total of 47 patients developed early postoperative complications, including 23 cases in the TG group and 24 cases in the EG group. The incidence of pulmonary infection was 22% (43/191), with 40 patients showing symptom improvement after treatment. However, one patient developed pleural effusion and received CT-guided thoracic puncture drainage and two patients developed respiratory failure due to lung infection and were transferred to ICU and discharged after improvement. One patient was transferred to ICU for pulmonary embolism and was discharged after improvement. One patient developed pulmonary small artery embolism after surgery, and was discharged after conservative treatment.

Three cases experienced postoperative bleeding but were all discharged after conservative treatment. Anastomotic fistula was confirmed in five patients by the reexamination of upper digestive tract angiography. This was improved after a treatment regimen including water fasting, anti-infection, unobstructed drainage, and enhanced nutrition. Postoperative acute cerebral infarction and atrial fibrillation occurred in two patients and one patient, respectively. These conditions improved after active treatment. There was no pyloric obstruction caused by pyloric spasm during hospitalization.

None of the patients experienced any recurrence or death during hospitalization, and no significant statistical difference was observed in the grading of complications between the two groups.

### 3.4 Long-term postoperative follow-up

**Figure 3:**
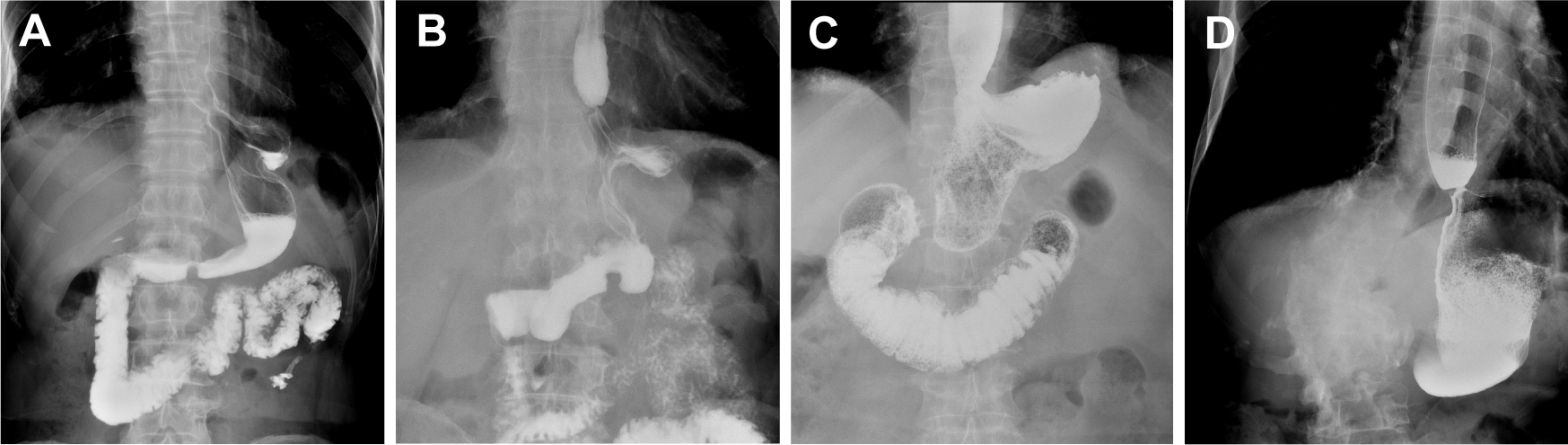
Typical contrast images of the upper digestive tract. **(A)** Upper digestive tract contrast images of normal tubular esophagogastric anastomosis; **(B)** Upper digestive tract contrast images for patients with anastomotic of stenosis tubular esophagogastric anastomosis; **(C)** Upper digestive tract contrast images of normal traditional esophagogastrostomy; **(D)** Upper digestive tract contrast images for patients with anastomotic stenosis of traditional esophagogastrostomy.

**Figure 4:**
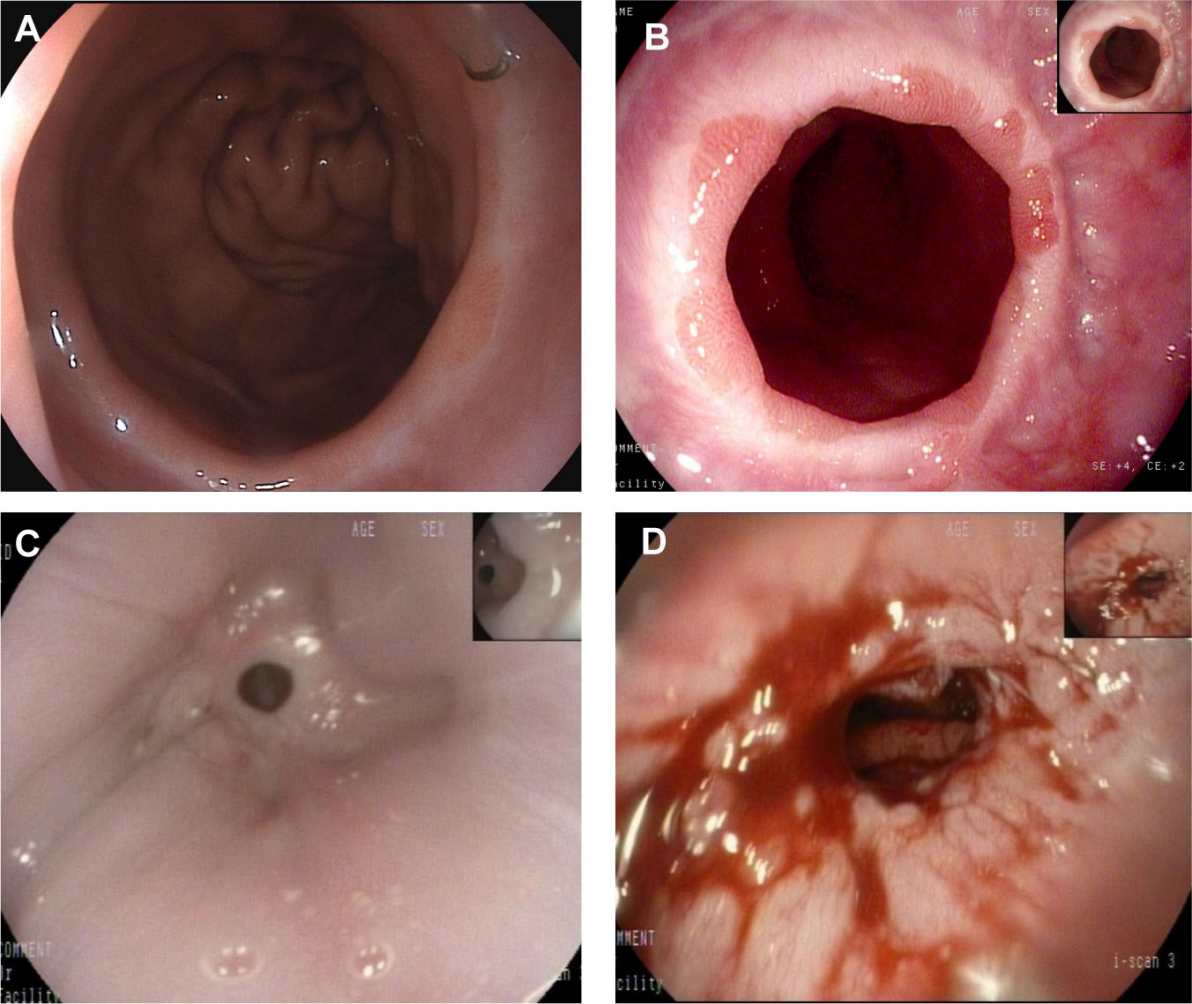
A representative gastroscopic image. **(A)** Normal anastomotic status of the patient after esophagogastric anastomosis; **(B)** Anastomotic status in patients with reflux esophagitis; **(C)** anastomotic stenosis; **(D)** Anastomotic status after endoscopic dilation.

The quality of life of patients was statistically compared in the 3rd month, 1st year and 2nd year after surgery. The analysis results are shown in **Tables 4**–**6**. Compared with the EG group, the average postoperative GredQ score of the TG group was lower, but with time, the score of the two groups gradually decreased. The anastomotic width of patients also showed a narrowing trend year by year, with statistically significant difference between the two groups (*p* < 0.05). At three months after surgery, the number of patients with anastomotic width less than 10 mm was significantly higher in the TG group than in the EG group (20% vs. 5%). In addition, the proportion of patients in TG group diagnosed with anastomotic stenosis after surgery (balloon dilation required) was higher than that in EG group (12% vs. 3%, *p* = 0.021). Notably, under the same dysphagia score, there were more patients in the TG group than in the EG group (*p* = 0.041). Typical postoperative images of the upper digestive tract and gastroscope are shown in **Figures 3**, **4**, respectively.

**Table 4:** Postoperative quality of life (3 months after surgery).

	TG group	EG group	P value
	n = 98	n = 93	
GredQ score	10.02±2.18	11.04±2.44	0.003
anastomotic width (mm)	13.51±4.06	15.15±3.24	0.002
anastomotic width less than 10mm	20 (20%)	5 (5%)	0.002
dysphagia score			0.041
0	69 (70%)	79 (85%)	
1	9 (9%)	8 (9%)	
2	8 (8%)	3 (3%)	
3~4	12 (12%)	3 (3%)	
dysphagia score > 2	12 (11%)	3 (3%)	0.021
anastomotic stenosis*	12 (12%)	3 (3%)	0.021

*Patients whose anastomotic width was less than 10mm and dysphagia score was more than 2 points were defined as anastomotic stenosis, and endoscopic anastomotic balloon dilation was performed.

**Table 5:** Postoperative quality of life (1 year after surgery).

	TG group	EG group	P value
	n = 98	n = 93	
GredQ score	8.91±2.43	10.01±2.58	0.003
reflux esophagitis			0.014
yes	19 (19%)	33 (36%)	
no	46 (47%)	27 (29%)	
not examined	33 (34%)	33 (36%)	
anastomotic width (mm)	13.21±2.72	14.18±2.27	0.008
anastomotic width less than 10mm	8 (8%)	3 (3%)	0.143
dysphagia score			0.327
0	73 (74%)	79 (85%)
1	14 (14%)	9 (10%)
2	9 (9%)	4 (4%)
3~4	2 (2%)	1 (1%)
dysphagia score > 2	2 (2%)	1 (1%)	1
anastomotic stenosis	2 (2%)	1 (1%)	1

**Table 6:** Postoperative quality of life (2 years after surgery).

	TG group	EG group	P value
	n = 91	n = 86	
GredQ score	8.31 ± 1.94	9.15 ± 2.01	0.005
reflux esophagitis			0.04
yes	12 (13%)	22 (26%)	
no	49 (54%)	32 (37%)	
not examined	30 (33%)	32 (37%)	
anastomotic width (mm)	12.70 ± 2.12	13.73 ± 1.86	< 0.001
anastomotic width less than 10mm	3 (3%)	0 (0%)	0.246
dysphagia score			0.683
0	87 (96%)	84 (98%)	
1-4	4 (4%)	2 (2%)	
dysphagia score > 2	0	0	–
anastomotic stenosis	0	0	–

For various reasons, not all patients received gastroscopy on time after surgery. We conducted statistical analysis of reflux esophagitis among the patients in the two groups who underwent regular gastroscopy, and the results are shown in **Table 7**. In total, 125 patients underwent gastroscopy 1 year after surgery, with reflux esophagitis confirmed in 19 patients (29%) in the TG group and 33 patients (55%) in the EG group. However, 2 years after surgery, gastroscopy confirmed reflux esophagitis in 12 patients (20%) in the TG group and 22 patients (41%) in the EG group, with statistically significant differences between the two groups (*p* < 0.05).

**Table 7:** Rate of reflux esophagitis.

	TG group	EG group	P value
reflux esophagitis (1 year) ^a^	19 (29%)	33 (55%)	0.003
reflux esophagitis (2 years) ^b^	12 (20%)	22 (41%)	0.013

a, TG group n=65; EG group n=60.

b, TG group n=61; EG group n=54.

### 3.5 ICG analysis

Among the 191 patients, 72 underwent ICG tracer-guided laparoscopic gastrectomy and 119 underwent conventional laparoscopic gastrectomy. After propensity score matching, 68 patients in the ICG group and 68 patients in the non-ICG group were included (**Table 8**). The two groups had similar digestive tract reconstruction time (58.31 ± 7.56 min vs. 59.63 ± 7.60min, *p* = 0.310]. Compared with the non-ICG group, the intraoperative blood loss in the ICG group was significantly lower (*p* = 0.007). For postoperative pathology, the number of lymph nodes obtained in ICG group (34.41 ± 6.15) was significantly higher than that in non-ICG group (30.69 ± 6.43) (*p* < 0.001). However, there was no significant difference in the number of positive lymph nodes between the two groups (*p* = 0.131).

**Table 8:** ICG analysis(After propensity matching).

	ICG group	Non-ICG group	P value
	(n = 68)	(n = 68)	
operation time(min)	222.35 ± 34.98	243.88 ± 40.90	0.001
lymph node dissection time(min)	111.62 ± 12.41	118.53 ± 12.03	0.001
digestive tract reconstruction time(min)	58.31 ± 7.56	59.63 ± 7.60	0.310
blood loss(ml)	34.85 ± 25.89	48.97 ± 33.60	0.007
number of lymph nodes	34.41 ± 6.15	30.69 ± 6.43	<0.001
number of positive lymph nodes	0.65 ± 1.87	1.31 ± 3.06	0.131

### 3.6 Survival analysis

**Figure 5:**
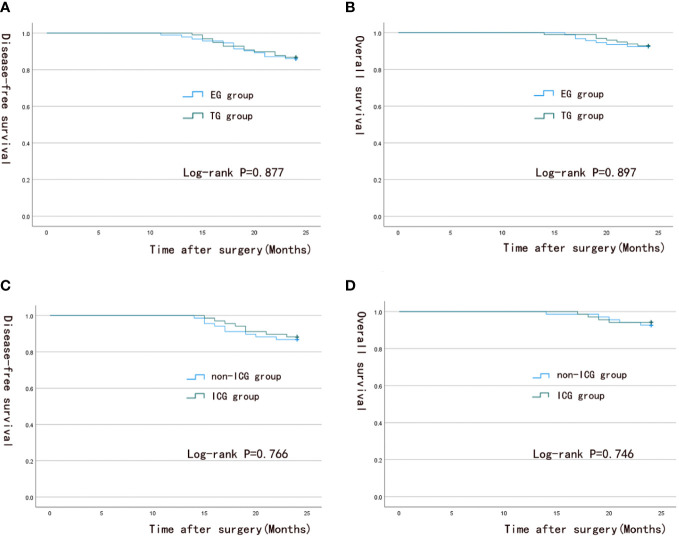
Survival analysis curve. **(A, B)** 2-year overall survival and 2-year disease-free survival in the TG group and EG group; **(C, D)** 2-year overall survival and 2-year disease-free survival in the ICG group and the non-ICG group.

The median postoperative follow-up time of patients was 24 months (range, 14-24 months). The survival analysis results are shown in **Figure 5**. At the end of the study, the two-year OS rates and DFS rates of patients were 92.9% and 86.7% in the TG group and 92.5% and 86% in the EG group (*p* = 0.897; 0.877), respectively. No significant difference was found in survival outcomes between the two groups. The overall OS rates and DFS rates were 94.1% and 88.2% for the ICG group and 92.6% and 86.8% for the non-ICG group (*p* = 0.746; 0.766), respectively. No significant difference was found in survival outcomes between the two groups.

## 4 Discussion

At present, radical proximal gastrectomy for early upper gastric cancer is widely accepted by doctors, but the optimal method for digestive tract reconstruction remains controversial (22, 23). At present, various reconstruction methods of digestive tract after proximal gastrectomy are used, mainly including traditional esophagogastrostomy, tubular esophagogastric anastomosis, double muscle flap anastomosis, jejunal interposition anastomosis, and double channel anastomosis. In tubular esophagogastric anastomosis, the residual stomach is cut into a tube and then anastomosed with the esophagus during proximal gastrectomy. Shiraishi reported (24) that a patient treated with tubular esophagogastric anastomosis achieved a total reflux time of 1 min within 12 h after surgery. In addition, the patient reported no reflux symptoms such as heartburn, retrosternal pain and acid reflux, indicating that tubular esophagogastric anastomosis has a good anti-reflux effect. Chen’s study also proved that tubular esophagogastric anastomosis has a lower incidence of early postoperative complications, which suggested that it is a safe and feasible method for digestive tract reconstruction (25).

Similarly, in the present study, both traditional esophagogastrostomy and tubular esophagogastric anastomosis after laparoscopic radical proximal gastrectomy were found to be safe and feasible. Compared to EG group, the TG group had less postoperative gastric tube drainage volume, shorter gastric tube drainage time, and higher first drinking time. Additionally, the reuse rate of PPIs was lower and the application time of PPIs was shorter in the TG group. Besides, compared with the EG group, the occurrence of postoperative esophagogastric reflux symptoms and postoperative life quality of patients in TG group were significantly improved, but the incidence of postoperative anastomotic stenosis was higher and the anastomotic width was narrower.

We also found no significant difference in the time spent performing digestive tract reconstruction between the two groups. This means that compared with traditional esophagogastric anastomosis, tubular esophagogastric anastomosis does not increase the difficulty of surgery. On the contrary, it decreases the operation time, reducing the possibility of intraoperative accidents and postoperative complications. Similar results were obtained by Chen (13). In this study, tubular esophagogastric anastomosis took longer time than traditional esophagogastrostomy (280.00 ± 25.48 min vs. 268.41 ± 28.18 min), but this time difference was no statistically significant.

In the present study, although the recovery time of gastrointestinal function was not significantly different between the two groups, the average daily gastric tube drainage volume of patients in the TG group was significantly lower compared with the EG group. As a result, the gastric tube removal time was shorter in the TG group. Gastric tube drainage volume and postoperative gastrointestinal function recovery are important factors for determining when patients can start drinking water. The results showed that the first drinking time of patients in the TG group was shorter compared with the EG group (121.03 ± 32.79 vs. 132.83 ± 29.24). As a result, the TG group had a shorter postoperative hospital stay than the EG group (9.43 ± 2.72 vs. 10.19 ± 2.99), with no statistically significant difference between the two groups (*p* = 0.066). The removal of most of the gastric in the lesser curvature of the stomach using tubular esophagogastric anastomosis and the location of gastric acid-secreting parietal cells in the lesser curvature of the stomach could explain this result. The procedure also decreased the number of parietal cells, which reduced gastric acid secretion and gastric tube drainage volume, thus achieving the goal of anti-reflux. Domergue’s study (26) monitored the PH of gastric fluid in patients with intrathoracic esophagogastric anastomosis for 24 hours and found that decreased gastric acid secretion after tubular esophagogastric anastomosis effectively reduced the incidence of reflux esophagitis. Our results also revealed that the PH of gastric fluid was higher in the TG group than in EG group (6.31 ± 0.50 vs. 6.13 ± 0.59, *p* = 0.022), indicating that tubular esophagogastric anastomosis reduced gastric acid secretion. One study also found that tubular esophagogastric anastomosis decreased gastric acid production by 30 percent (27).

In the present study, the average incidence of early postoperative complications was 25%, including 23% in the TG group and 26% in the EG group. The early complication rate of Clavien-Dindo classification II and above was 21%, with no statistically significant difference between the two groups. Common perioperative complications after proximal gastrectomy include pulmonary infection, hemorrhage, anastomotic fistula and pyloric obstruction. Among them, gastric postoperative pyloric obstruction is of a particular concern because it causes a sense of fullness after eating, vomiting and even aspiration pneumonia in severe cases, which seriously affect the patient’s postoperative quality of life. The main methods used to treat and prevent postoperative pyloric obstruction include pyloroplasty, pyloric myotomy and mechanical dilation of the pyloric site, before or during surgery, and postoperative balloon dilation under symptomatic endoscopic conditions (28–30). Nienhuser’s research also showed that mechanically dilating the pylorus can significantly reduce the incidence of delayed gastric emptying (31). During the operation, we used mechanical dilation of pyloric muscle, which effectively prevented pyloric obstruction caused by pyloric spasm. No pyloric stenosis occurred in all patients after the operation. Zhu’s study also demonstrated that mechanical pyloric dilation during proximal gastrectomy can prevent pyloric stenosis due to postoperative pyloric spasm (32).

The anti-reflux effect of various digestive tract reconstruction methods after proximal gastrectomy has been the focus of research. Multiple retrospective studies (13, 33) have revealed that the incidence of reflux esophagitis in traditional esophagogastrostomy and tubular esophagogastric anastomosis is about 22%-28.6% and 5.7%-10.7%, respectively. Many scholars hold the view that tubular esophagogastric anastomosis is superior to traditional esophagogastric anastomosis, and our findings are consistent with those from previous reports (34). This study demonstrated that the incidence of endoscopically confirmed reflux esophagitis after tubular esophagogastric anastomosis was lower than that of conventional esophagogastric anastomosis. GerdQ score was used to evaluate patients’ long-term quality of life at 3 months, 1 year and 2 years after surgery, and the results indicated that the score of patients in the TG group was lower compared with that of patients in the EG group, suggesting that tubular esophagogastric anastomosis has a better anti-reflux effect. This was mainly ascribed to the possibility that tubular esophagogastric anastomosis on one hand reduces the secretion of gastric acid, prolongs reflux distance, and makes it difficult for gastric acid to reflux to the esophagogastric anastomosis. On the other hand, the tubular structure of the residual stomach is more consistent with the physiological pipeline, which reduces the contents of the residual stomach thereby promoting gastric emptying to consolidate the anti-reflux effect. A study measuring the gastric emptying rate after esophagogastric anastomosis confirmed that the emptying rate of food in the whole stomach is lower than that in the tubular stomach, and the narrower the tube, the faster the emptying rate (35). Elsewhere, it was reported that measuring the pressure in the gastric tube after proximal gastrectomy tubular esophagogastric anastomosis showed that the movement of the distal gastric tube in patients with reflux esophagitis was significantly lower than that in patients without reflux esophagitis (36), indicating that the occurrence of reflux esophagitis is somewhat related to the movement of the gastric tube, which also supported our speculation. Notably, after proximal gastrectomy, the gastrointestinal motor function gradually recovered over time during the interdigestive period, which decreased the incidence of reflux esophagitis 1 year after surgery compared with 1 month after surgery, as well as reduced the grading. Toyomasu’s research confirmed this finding (37). However, in our study, both the incidence of postoperative reflux esophagitis and postoperative GredQ score showed a decreasing trend in both groups, indicating that the severity of gastroesophageal reflux was alleviated over time.

In addition, in the tubular stomach prepared in our laboratory, the esophagogastric anastomosis is 5cm away from the upper end of the residual stomach, forming a structure similar to that of the stomach fundus allowing restoration of the physiological structure of the stomach as far as possible. It therefore provides a cushioning effect on the reflux of stomach contents, alleviating the discomfort in patients and exerting anti-reflux effect. A study by Ueda (38) reported a similar concept, and suggested that the tubular stomach anastomosis creates a long and narrow stomach duct, as well as a shape similar to the angle of the stomach, which prevents acid reflux.

PPIs are routinely administered until the patient’s first drinking during the period of postoperative diet prohibition. Some patients may show intolerable gastrointestinal reactions such as acid reflux, heartburn and nausea after withdrawal of the drug. In this case, PPIs were applied to improve symptoms. Among them, the postoperative PPIs reuse rate of TG group was 6%, and the postoperative PPIs application time was 5.46 ± 1.98 day; the postoperative PPIs reuse rate of EG group was 23%, and the postoperative PPIs application time was 6.44 ± 2.25 day, which demonstrated the advantages of tubular esophagogastric anastomosis in anti-reflux. During the follow-up, we found that most patients took the PPIs when they had symptoms of reflux. They reported that the PPIs were effective in relieving discomfort such as acid reflux and heartburn, and there were no side effects from long-term use. Numerous studies have confirmed the beneficial effects of the PPIs in this situation (39). A randomized trial study confirmed a significant reduction in gastric acid production and the incidence of reflux esophagitis after the PPIs treatment (40).

We instructed patients to undergo upper digestive tract angiography 3 months after surgery to measure the width of the anastomosis and observe the status of the anastomosis. From 3 months to 2 years after surgery, the postoperative anastomotic width of the two groups showed a decreasing trend, and the incidence of anastomotic stenosis in the TG group was 12%, much higher than that in the EG group (3%), which revealed a significant difference between the two groups. At the 3rd month after surgery, the difference in dysphagia score between the two groups was statistically significant. The medical intervention effectively improved dysphagia and anastomotic stenosis. Although some studies have found no significant difference in the incidence of anastomotic stenosis between traditional esophagogastric anastomosis and tubular esophagogastric anastomosis (15.4% vs. 15.1%) (41), we found that there were still significant differences in the incidence of anastomotic stenosis between the two groups. Many patients reported varying degrees of eating obstruction or only liquid diet during the postoperative recovery period. Although most patients could stimulate spontaneous anastomotic dilation through diet, some patients required endoscopic balloon dilation before resumption of normal diet. Theoretically, the risk factors of anastomotic stenosis are influenced by anastomotic tension, scar contraction, the influence of radiotherapy or chemotherapy, anastomotic leakage, gastroesophageal reflux and other factors (42). Because tubular esophagogastric anastomosis requires more closure apparatus, it produces more surgical scars compared with traditional esophagogastric anastomosis, which is one of the common causes of anastomotic stenosis. Therefore, during the design of tubular gastric, we performed anastomosis at the greater curvature of the stomach 5cm away from the top of the residual stomach to minimize the influence of surgical scar contraction on the width of the anastomosis. But this effect need to be further studied. Our study also confirmed that although the incidence of anastomotic stenosis was increased after tubular esophagogastric anastomosis, most patients resumed normal diet after endoscopic balloon dilation therapy, and hence did not cause lifelong disease burden to patients. Notably, although some patients overcame dysphagia after endoscopic balloon dilation, they gradually developed symptoms of reflux esophagitis in the later stage.

In the present study, there was no significant difference in 2-year OS and 2-year DFS between the two groups. In addition, no significant difference in postoperative pathological T stage and N stage was observed between the two groups of patients, and there was no significant statistical differences were recorded in the preoperative basic clinical data. Moreover, the 2-year OS and 2-year DFS were comparable between the two groups. We believe that patients have a better prognosis due to the clinicopathologic staging of the patients. Jiao’s study postulated that advanced pathology was the main cause of poor prognosis of patients (43). However, the patients in our study had relatively early pathological stages, with the proportion of early gastric cancer in TG group and EG group accounting for 58% and 55% respectively, and the positive rate of postoperative lymph node was 26% and 22%, respectively. The depth of tumor invasion was relatively shallow, and the rate of lymph node metastasis was low. Thus, the tumor burden of patients was small resulting in good prognosis.

In the analysis of the ICG group after propensity score matching, we found that the total operation time and lymph node dissection time in the ICG group were significantly shorter relative to those in the non-ICG group, which was ascribed to the tracer and navigation effect of ICG on lymph nodes. The ICG group also had lower intraoperative blood loss compared to the non-ICG group, and this effect was closely related to the ability of ICG to distinguish lymphatic vessels. Since ICG fluorescent dye can reach lymph nodes around the lesion through lymphatic drainage, lymph nodes, peripheral blood vessels or other tissues can be easily detected by switching common mode into fluorescence mode during the operation, thus improving the efficiency of the operation and shortening the operation time. In addition, ICG can remain in lymph nodes for a certain period of time, which improves the removal of lymph nodes during surgery, and also conducive to the sorting of lymph nodes in the pathological specimens of patients *in vitro*, so that the number of lymph nodes obtained in the ICG group is more than that in the non-ICG group. Our previous study (17) demonstrated that ICG labeling significantly reduced the time required for laparoscopic free dissection, and the total number of lymph nodes obtained after surgery was significantly higher compared with that of the non-ICG group. Other studies have suggested that the use of ICG in laparoscopy may have clinical benefits (44). In summary, the application of ICG in the auxiliary role of laparoscopic surgery has many merits.

Nevertheless, this research still leaves much to be explored. Since this is a single-center retrospective study with a small sample size, more patients should be included in subsequent studies or multi-center studies to improve the level of evidence. Given that the anastomotic width was measured under the upper digestive tract imaging, some bias is inevitable, and an effective approach to measure the esophagogastric anastomosis needs to be further explored. In addition, our center also carried out the operation of double-tract anastomosis, but the number of patients with double-tract anastomosis was too small to reach the ideal number of cases by the end of follow-up time, thus we could not perform the analysis. In future, we will continue to study the advantages and disadvantages of the operation of tubular esophagogastric anastomosis and double-tract anastomosis.

## 5 Conclusion

In conclusion, the present results demonstrate that laparoscopic radical proximal gastrectomy with tubular esophagogastric anastomosis is safe and feasible, and its anti-reflux effect is significantly better compared with that of traditional esophagogastric anastomosis. Despite the high incidence of postoperative anastomotic stenosis, it has little impact on patients’ long-term quality of life after clinical intervention. It is recommended for digestive tract reconstruction after proximal gastrectomy. The application of ICG tracer technique in laparoscopic proximal gastrectomy has some clinical benefits.

## Data availability statement

The original contributions presented in the study are included in the article/**Supplementary Material**. Further inquiries can be directed to the corresponding author.

## Ethics statement

The studies involving human participants were reviewed and approved by Medical Ethics Committee of Qilu Hospital of Shandong University. The patients/participants provided their written informed consent to participate in this study. Written informed consent was obtained from the individual(s) for the publication of any potentially identifiable images or data included in this article.

## Author contributions

YW-B is the corresponding author. WM and HY-D are joint first authors. WM, HY-D designed the study, collect relevant pictures, and prepared the manuscript. YW-B, WM, CW-B, ZR-D conducted the laparoscopic radical proximal gastrectomy. HY-D, CZ-W, OY J, LY-Z, YZ-P, CC-Q conducted the data collection and analysis and long-term patient follow-up. SD-P provided relevant inputs for the discussion. YW-B, WM conduct quality control. All authors contributed to the preparation of this article and approved the submitted version.
