# Supplementary material for: Clinical effect and follow-up of laparoscopic radical proximal gastrectomy for upper gastric carcinoma
Source: Front Oncol. 2023 Mar 29;13:1167177. doi: 10.3389/fonc.2023.1167177 (PMC10090458; doi:10.3389/fonc.2023.1167177)
Supplement: Supplementary file 1 [file Table_1.docx]

Supplementary Material

Clinical effect and follow-up of laparoscopic radical proximal gastrectomy for upper gastric carcinoma

**WEI Meng, HUANG Ya-di1, CAO Wei-bo, ZHAO Ru-dong, CHENG Ze-wei, OU Yang-jun, YAN Ze-peng, CHEN chuan-qi, LIANG Yi-ze, SUN Dan-ping, YU Wen-bin***

*** Correspondence:** Corresponding Author: YU Wen-bin, Department of Gastrointestinal Surgery Qilu Hospital of Shandong University, Jinan, Shandong province, 250000, China
E-mail: wenbin_yu2003@163.com

## Supplementary Tables

### Supplementary table 1

**Classification of Surgical Complications**

| **Grade** | **Definition** |
| --- | --- |
| Grade I | Any deviation from the normal postoperative course without the need for pharmacological treatment or surgical, endoscopic, and radiological interventions  Allowed therapeutic regimens are: drugs as antiemetics, antipyretics, analgetics, diuretics, electrolytes, and physiotherapy. This grade also includes wound infections opened at the bedside |
| Grade II | Requiring pharmacological treatment with drugs other than such allowed for grade I complications  Blood transfusions and total parenteral nutrition are also included |
| Grade III  Grade IIIa  Grade IIIb | Requiring surgical, endoscopic or radiological intervention  Intervention not under general anesthesia  Intervention under general anesthesia |
| Grade IV  Grade IVa  Grade IVb | Life-threatening complication (including CNS complications)* requiring IC/ICU management  Single organ dysfunction (including dialysis)  Multiorgan dysfunction |
| Grade V | Death of a patient |

*Brain hemorrhage, ischemic stroke, subarrachnoidal bleeding, but excluding transient ischemic attacks.

CNS, central nervous system; IC, intermediate care; ICU, intensive care unit.

### Supplementary table 2

**The GerdQ questionnaire**

| **Question** | **Frequency score (points) for symptom** | | | |
| --- | --- | --- | --- | --- |
|  | **0 day** | **1 day** | **2-3days** | **4-7days** |
| How often did you have a burning feeling behind your breastbone (heartburn)? | 0 | 1 | 2 | 3 |
| How often did you have stomach contents (liquid or food) moving upwards to your  throat or mouth (regurgitation)? | 0 | 1 | 2 | 3 |
| How often did you have pain in the center of the upper stomach? | 3 | 2 | 1 | 0 |
| How often did you have nausea? | 3 | 2 | 1 | 0 |
| How often did you have difficulty getting a good night’s sleep because of your heartburn  and/ or regurgitation? | 0 | 1 | 2 | 3 |
| How often did you take additional medication for your heartburn and/or regurgitation,  other than what the physician told you to take? (such as T ums, Rolaids, Maalox?) | 0 | 1 | 2 | 3 |

### Supplementary table3

**Dysphagia score**

| **score** | **Definition** |
| --- | --- |
| 0 | able to consume a normal diet |
| 1 | able to eat some solid food |
| 2 | able to swallow only semisolid food |
| 3 | able to swallow liquids only |
| 4 | unable to swallow liquids |
